# Experience in the Management of Vaginal Cuff Dehiscence and Evisceration: A Retrospective 37-Year Single-Center Study

**DOI:** 10.3389/fsurg.2022.880875

**Published:** 2022-05-13

**Authors:** Xiao Ma, Dong-Yan Cao, Yu-Xin Dai

**Affiliations:** ^1^ Department of Obstetrics and Gynecology, Peking Union Medical College Hospital, Chinese Academy of Medical Sciences & Peking Union Medical College, Beijing, China; ^2^ National Clinical Research Center for Obstetric & Gynecologic Diseases, Beijing, China

**Keywords:** hysterectomy, laparoscopic hysterectomy, vaginal cuff dehiscence and evisceration, manual reduction, transvaginal cuff closure

## Abstract

**Purpose:**

Vaginal cuff dehiscence (and evisceration) (VCD(E)) is an extremely rare and late-onset complication of total hysterectomy (TH). Limited evidence is available to guide clinicians in managing VCD(E). This study aimed to summarize the clinical characteristics of patients with VCD(E) treated in our center and share our experience in managing VCD(E).

**Patients and methods:**

From 1983 to 2020, a total of 14 cases of VCD(E), including 10 cases in our hospital and 4 cases in other hospitals, were included. Medical records were reviewed to summarize the clinical features and management of VCD(E).

**Results:**

The incidence of VCD(E) in our hospital was 10/46,993 (0.02%), and all 10 patients underwent laparoscopic hysterectomy. The median TH-to-VCD(E) interval was 3.13 months (8 days–27.43 months), and 11/14 (78.57%) patients experienced VCD(E) after coitus. The 3 major symptoms included abdominal pain in 11 patients, irregular vaginal bleeding in 8, and sensation of bulging or prolapsed organs in 4. Except for 2, most patients presented to our hospital within 72 h since the onset of the discomfort. All 14 cases were diagnosed through speculum examination: 3 had simple VCD, and 11 had VCDE. The protruding bowels of 4 patients were immediately manually repositioned in the emergency department without anesthesia. Regarding the surgical approach, 11 patients underwent simple transvaginal, 2 patients underwent laparoscopic-vaginal combined (transvaginal cuff closures), and 1 patient underwent laparoscopic. All but 1 patient did not undergo resection of the eviscerated organs. The median follow-up period was 39.33 (7.9–159.33) months. No patients showed any evidence of recurrence to date.

**Conclusions:**

Laparoscopic hysterectomy is a risk factor for VCD(E), and early initiation of sexual intercourse is the most common trigger of VCD(E). Clinicians should educate patients to postpone sexual intercourse for at least 3–6 months after TH. Immediate medical attention and patient-specific surgical management are crucial to avoid serious complications.

## Introduction

Total hysterectomy (TH) is one of the most common gynecological procedures (1) and is widely applied in various benign and malignant diseases. Genitourinary and gastrointestinal tract injury, bleeding, infection, and vaginal cuff dehiscence (VCD) are the most common complications of TH (2). In contrast to other complications of TH, VCD is an extremely rare and late-onset complication. VCD refers to the partial or full-thickness separation of the vaginal cuff (3). It is termed vaginal cuff dehiscence and evisceration (VCDE) when the intraperitoneal contents protrude from the cuff defect (4). The small bowel is the most frequently eviscerated organ, but evisceration of other organs, such as the sigmoid colon, greater omentum, appendix, and adnexa uteri (5), have also been reported. VCD is a rare and life-threatening condition that may lead to evisceration. The most common clinical presentations of VCD(E) are sudden lower abdominal pain and irregular vaginal bleeding. Patients with evisceration may also feel a bulging sensation or find the prolapsed organs.

Due to its low incidence, most reported cases are sporadic. Numerous studies have focused on the risk factors for VCD(E) and strategies to decrease its morbidity. Limited evidence is available to guide clinicians in managing VCD(E). Herein, we describe the clinical characteristics and management of 14 cases of VCD(E) treated in our hospital and share our original experience in the treatment of VCD(E).

## Materials and Methods

We searched and retrieved the medical records of patients who underwent TH and who were diagnosed with “VCD(E)” from 1983 to 2020 in the database of Peking Union Medical College Hospital (PUMCH). Data extracted from the medical records mainly included demographic and clinical characteristics of patients, details of hysterectomy and VCD(E), and management of VCD(E). Follow-up information was collected from the clinical database of PUMCH or through telephone interviews with patients. This study was approved by the Ethics Committee of PUMCH. Categorical variables are expressed as numbers and proportions, while continuous variables are described as medians and ranges. Statistical analysis was performed using SPSS version 25.0 (IBM SPSS Statistics).

## Results

### Patient Characteristics

From 1983 to 2020, 46,993 patients underwent TH at PUMCH, and 10 (0.02%) experienced VCD(E) after laparoscopic hysterectomy (LH). In addition, 4 patients who had TH in other hospitals also experienced VCD(E) and were treated at PUMCH during this period. Therefore, 14 patients with VCD(E) were identified from the PUMCH database, and their medical records were reviewed. Demographic data of patients are detailed in Table 1. The patients’ median age was 46 years (31–55 years). The median body mass index (BMI) was 23.53 kg/m^2^ (18.29–25.71 kg/m^2^); 6 (42.86%) patients were overweight but not obese according to Chinese standards, and 2 of them had diabetes mellitus.

**Table 1:** Demographic data of patients.

Case No.	Age(yr)	Parity	Delivery	BMI (kg/m^2^)	Comordity
1	48	1	CS	20.14	Breast cancer(had adjuvant therapy)
2	52	2	VD	24.09	DM
3^a^	54	1	VD	18.90	No
4	52	1	CS	18.29	No
5^a^	44	1	VD	21.01	No
6^a^	31	1	CS	21.57	No
7	50	1	VD	24.09	No
8	42	1	VD	23.23	No
9	53	1	VD	24.22	No
10	55	1	VD	25.22	DM, HTN, Hyperlipidemia
11^a^	44	1	VD	24.22	No
12	39	0	/	20.96	No
13	44	1	CS	25.71	Breast cancer(had adjuvant therapy)
14	40	2	CS	23.83	No

*VD, vaginal delivery; CS, cesarean section; BMI, body mass index; DM, diabetes mellitus;HTN hypertension.*

*^a^* *Patients who underwent TH in other hospitals.*

### Details of Total Hysterectomy

**Figure 1:**
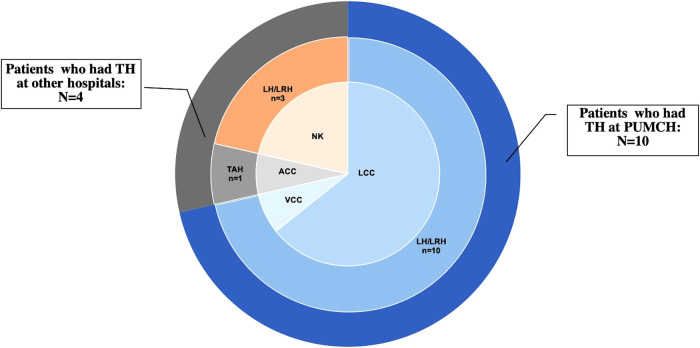
The distribution of different types of TH and cuff closure.

Three (21.43%) patients were menopausal before TH, and 6 (42.86%) underwent bilateral or unilateral oophorectomy during TH. Moreover, 5 patients received TH for benign diseases, 3 for precancerous lesions, 5 for malignancies, and 1 for prophylaxis and myoma. Among the 14 patients, 5 had a history of adjuvant therapy (3 of them for gynecological malignancy, and 2 of them for breast cancer). Regarding the surgical approach, only 1 patient (7.14%) underwent laparotomy with transabdominal cuff closure, 13 cases (92.86%) underwent laparoscopy, including transvaginal cuff closure (VCC) in 1 and laparoscopic cuff closure in 9; the other 3 were unknown (Figure 1 and Table 2). All the 10 patients who had LH at PUMCH underwent cuff closure with absorbable and continuous sutures. Information about TH in our study is presented in Table 2.

**Table 2:** Information about TH.

Case No.	Indication	Menopause	Mode of hysterectomy	Additional procedure	Duration of operation(min)	EBL(mL)	Route of cuff closure	Type of suture	Suturing method	Intraoperative complication	Postoperative complication		Adjuvant therapy
											Anemia	Fever	
1	Atypical endometrial hyperplasia	Spontaneous menopause	LH	BSO	40	20	LCC	VCP358H	Simple running	No	Mild	No	None
2	Adenomyosis + Myoma	No	LH	BS	60	30	LCC	VCP518H	Simple running	No	Mild	No	None
3^a^	Ovarian Cyst	No	TAH	BSO	NK	NK	ACC	NK	NK	NK	NK	NK	None
4	Myoma + Ovarian Cyst	No	LH	BS + UO	90	40	LCC	VCP358H	Simple running	No	Mild	No	None
5^a^	Adenomyosis	No	LH	BS	NK	NK	NK	NK	NK	NK	NK	NK	None
6^a^	Cervical cancer	No	LRH	BS + LND + BOS	NK	NK	NK	NK	NK	NK	NK	NK	Chemotherapy and brachytherapy
7	Complex endometrial hyperplasia + Ovarian Cyst	No	LH	BS + Removal of ovarian cyst	60	50	LCC	VCP358H	Simple running	No	Moderate	No	None
8	Myoma	No	LH	BS	55	50	LCC	VCP358H	Simple running	No	Mild	No	None
9	CIN + Myoma	Spontaneous menopause	LH	BSO	95	50	LCC	VCP518H	Simple running	No	NK	No	None
10	Endometrial cancer	Spontaneous menopause	LH	BSO	85	20	LCC	VCP518H	Simple running	No	No	No	Brachytherapy
11^a^	Cervical cancer	No	LRH	BS + LND + BOS	NK	NK	NK	NK	NK	NK	NK	NK	None
12	Cervical cancer	No	LH	None	100	NK	VCC	1-0 Dexon	Interlock	No	Mild	No	None
13	Myoma + Prophylactic	No	LH	BSO	70	10	LCC	VCP518H	Simple running	No	NA	No	None
14	Cervical cancer	No	LRH	BS + LND + BOS	210	400	LCC	VCP518H	Simple running	No	Moderate	Yes	Chemotherapy and brachytherapy

*TH, total hysterectomy; CIN, cervical intraepithelial neoplasia; LH, laparoscopic hysterectomy; TAH, transabdominal hysterectomy; LRH, laparoscopic radical hysterectomy; NK, not known; BS bilateral salpingectomy; BSO, bilateral salpingo-oophorectomy; UO, unilateral oophorectomy; LND, lymph node dissection; BOS, bilateral ovarian suspension; EBL, estimated blood loss; LCC, laparoscopic cuff closure; ACC, transabdominal cuff closure; VCC, transvaginal cuff closure.*

*^a^* *Patients who underwent TH in other hospitals.*

### Clinical Features and Management of VCD(E)

As shown in Table 3, the median TH-to-VCD(E) interval was 3.13 months (8 days–27.43 months). Eleven (78.57%) patients noted VCD(E) to be triggered by intercourse, 1 patient (7.14%) noted VCD(E) during brachytherapy and was asymptomatic, and the other 2 (14.28%) noted VCD(E) to be caused by strenuous activity. In our series, the 3 major symptoms, presenting separately or concurrently, included lower abdominal pain in 11, irregular vaginal bleeding in 8, and sensation of bulge or prolapsed organs in 4. All 14 cases were diagnosed through speculum examination: 3 cases had simple VCD, while 11 cases had VCDE. The prolapsed organs in 11 cases of VCD(E) included the bowel in 9 (81.82%) and omentum in 2 (18.18%). Since the onset of discomfort, most patients presented to PUMCH in a timely manner, with 9 patients presenting within ≤24 h (7 patients ≤6 h), 3 in >24 but ≤72 h, and 2 patients after >72 h. One patient experienced abdominal pain after intercourse, which spontaneously resolved approximately 3 h later. On the 11th day after the intercourse, she noticed a prolapsed bowel and was referred to the hospital. The other patient visited PUMCH on the 8th day after she experienced acute abdominal pain and found prolapsed tissue after coitus.

**Table 3:** Clinical features and management of VCD(E).

Case No.	TH-to-VCD(E) interval(d)	Trigger events	Onset-to-consultation interval	Symptoms	Anemia	Evisceration	Reposition prolapsed organs	Surigical interventions	Type of suture	Suturing method	Additional procedures	Antibiotics	Follow-up(d)	Recurrence
1	84	Postcoitus	6 h	Lower abdominal pain + found the prolapsed bowel	No	Bowel	UA	LCC	VCP358H	Figure-of-eight	/	Yes	791	No
2	102	Postcoitus	11 d	Lower abdominal pain	No	Bowel	UA	VCC	VCP358H	Simple running	/	Yes	237	No
3^a^	324	Postcoitus	1.5 h	Irregular vaginal bleeding + Lower abdominal pain	No	Bowel	WoA	VCC	VCP358H	Interlock	/	Yes	0	Lost of follow-up
4	80	Postcoitus	10.5 h	Lower abdominal pain + sensation of bulge	No	Bowel	UA	VCC	VCP603H	Interlock	/	Yes	884	No
5^a^	63	Postcoitus	1 h	Irregular vaginal bleeding + Lower abdominal pain + nausea and vomiting	No	Bowel	WoA	VCC	VCP358H	Interlock	Take a swab for culture(−)	Yes	1,219	No
6^a^	823	Postcoitus	5.5 h	Irregular vaginal bleeding + Lower abdominal pain	No	Bowel	UA	VCC	VCP518H	Figure-of-eight + interrupted	MBT of the bladder to consume bladder integrity preoperatively	Yes	1,107	No
7	135	Strenuous activity	2 d	Irregular vaginal bleeding + Lower abdominal pain	No	No	/	VCC	VCP358H	Interrupted	/	Yes	464	No
8	79	Postcoitus	1 d	Irregular vaginal bleeding	No	Omentum	UA	VCC	VCP518H	Interrupted	Take a swab for culture(-)+place a vaginal drainage-tube	Yes	2,431	No
9	144	Postcoitus	0.5 h	Irregular vaginal bleeding + Lower abdominal pain	No	Bowel	WoA	VCC	VCP518H	Interlock	/	Yes	2,441	No
10	50	During radiotherapy	1 d	Asymptomatic	No	No	/	VCC + exploratory laporoscopy	VCP358H	Interlock	MBT of the bladder to consume bladder integrity intraoperatively	Yes	814	No
11^a^	105	Postcoitus	2 h	Lower abdominal pain + found the prolapsed bowel	No	Bowel	WoA	VCC	1B-401	Simple running	Creatinine level of drainage	Yes	0	Lost of follow-up
12	8	Strenuous activity + straining on stool	12 h	Irregular vaginal bleeding + dizziness and cold sweat	Mild	No	/	VCC	1-0 Dexon	Simple running	Blood transfuison(10U+ 1,200 mL)	Yes	4,763	No
13	86	Postcoitus	6 h	Irregular vaginal bleeding + Lower abdominal pain + vaginal discharge	No	Bowel	UA	VCC	VCP358H	Interlock	/	Yes	3,480	No
14	770	Postcoitus	8 d	Lower abdominal pain + found the prolapsed bowels + vaginal discharge	No	Omentum	UA	VCC + laparocopy(partial omentectomy + BO)	VCP603H	Interlock	Pathological examination	Yes	644	No

*VCD(E), vaginal cuff dehiscence (and evisceration); TH, total hysterectomy; UA, Under anesthesia; WoA, without anesthesia; LCC, Laparoscopic cuff closure; VCC, transvaginal cuff closure; BO, bilateral oophorectomy; MBT, methylene blue test.*

*^a^Patients who underwent TH in other hospitals.*

Protruding bowels in 4 patients were manually repositioned without anesthesia in the emergency department. Then, they underwent cuff closure under anesthesia in the operating room. In our institution, 11 patients underwent a simple transvaginal approach, 2 underwent a combined laparoscopic-vaginal approach, and 1 underwent a laparoscopic approach. Of the 11 patients with VCDE, only one patient underwent partial resection of the eviscerated omentum because of necrosis. Different surgeons chose different sutures and suturing methods, in which absorbable antibacterial Vicryl sutures (VCP358H and VCP518H) and continuous sutures (simple running or interlock) were predominantly used. Oral or intravenous prophylactic antibiotics were administered perioperatively to each patient to avoid infection. More details are provided in Table 3.

### Follow-up

Two patients were lost to follow-up. The median follow-up period was 39.33 months (7.9–159.33 months). Eleven patients had an unremarkable postoperative course. One patient complained of vaginal spotting on postoperative day 11. She was identified as having partial knot slippage on the right side of the vaginal stump. Fortunately, the patient healed well after hemostasis and activity restriction. To date, there is no evidence of recurrence.

## Discussion

### Risk Factors

Surgical wound dehiscence is a potential complication post-surgery. A conceptual framework underlying surgical wound dehiscence has been developed (6), comprising physiological factors (comorbidities and lifestyle) and mechanical (intraoperative and postoperative) factors. Given the unique functional and anatomical characteristics of the vagina, VCD(E) is a special type of surgical wound dehiscence. We categorized the risk factors for VCD(E) into three groups: external force on the vaginal cuff, patient factors (physiological or pathological), and TH-related factors. Vaginal trauma during coitus is the most common trigger of VCD(E) (1, 4). Transvaginal procedures can also lead to VCD(E), including transvaginal ultrasonography (7) and intracavitary brachytherapy (8). Patient factors include three aspects (9): (1) factors impairing stump healing such as infection, hematoma, low estrogen status, glucocorticoid use, and immunosuppressant use; (2) increased abdominal pressure, such as in coughing, constipation, and strenuous activity; and (3) other factors, including BMI, age, and smoking. TH-related factors are mainly the method of colpotomy, type of vaginal cuff closure, and adjuvant therapy after TH. Most factors that affect wound healing are universal, but some risk factors differ between VCD(E) and other surgical wounds. For instance, obesity, high BMI, and old age are risk factors for surgical wound dehiscence but are protective factors against VCD(E) (10). A reasonable explanation is that elderly women are more likely to have lower estrogen levels and less sexual activity (11). We speculated that obese women with a higher BMI have a better buffering force against vaginal trauma during coitus than thin women with a normal or lower BMI. In our series, 9 patients were ≤50 years of age, and none were obese. It is important to note that 11 patients developed VCD(E) post-coitally, and 2 patients developed VCD(E) after strenuous activity. Two patients had comorbid diabetes mellitus, and VCDE was found in one of these two during brachytherapy. Five patients received adjuvant therapy for malignancy. Each patient in our study had at least 1 risk factor for VCD(E). Multiple factors contribute to VCD(E) (12) and having more risk factors compounds a patient’s risk of VCD(E).

Being one of the most common gynecological interventions, TH can be performed in various ways. According to the surgical route of the procedure, TH is divided into transabdominal hysterectomy, vaginal hysterectomy, laparoscopic-assisted vaginal hysterectomy, laparoscopic hysterectomy (LH), and robotic hysterectomy. VCD(E) is possible from any type of TH, but its prevalence varies widely depending on the TH route (13). The influence of the surgical route and type of cuff closure on the incidence of VCD(E) has been discussed with conflicting theories. Many studies (10, 14) have pointed out a possibly higher rate of VCD(E) after minimally invasive procedures than after transabdominal hysterectomy and vaginal hysterectomy. Electrosurgical thermal energy and techniques, magnifying effects, shallow tissue bites (15), poor knot integrity (16), low surgeon laparoscopic expertise (17), and prolonged inflammatory phase (18) are responsible for the increased risk of VCD(E) in minimally invasive surgeries. In our study, 10 of 14 VCD(E) cases underwent TH at PUMCH, and all of these 10 cases had undergone laparoscopy. In other words, out of 46,993 cases of TH in PUMCH, only 10 cases of LH experienced VCD(E) postoperatively, suggesting that LH was associated with a higher rate of VCD(E). To date, VCD(E) remains a rare event. Laparoscopic techniques have undergone rapid development over the last 30 years. Although some patient factors attributed to VCD(E), including age and low estrogen status, cannot be improved perioperatively, we still believe that optimal perioperative management and improvement of surgical skills are of greater value to the prevention of VCD(E).

### Clinical Features

The clinical presentation of VCD(E) could be abdominal pain, vaginal bleeding, vaginal discharge, and a sensation of a bulge yet free from any discomfort (4). Notably, evisceration could be observed in approximately one-third to more than half of VCDs, and up to 30% of VCD(E)s eventually undergo bowel resection. The severity of VCD(E) depends on the eviscerated organs, condition and duration of evisceration, and whether it is accompanied by serious complications, such as ileus, bowel necrosis, peritonitis, shock (hemorrhagic or septic), pneumoperitoneum (19), and pneumomediastinum (20). Of the 14 cases, 11 (78.57%) were accompanied by evisceration. Necrosis of the omentum only progressed in one case, and it was due to a late medical consultation. To address omentum necrosis, doctors performed partial omentectomy by laparoscopy. Nine of the 11 patients with VCDE had bowel protrusion from the split cuff. With timely medical consultation and intervention, none of the 9 patients developed incarceration, ischemia, or necrosis, and they all recovered without the need to remove the prolapsed bowel.

### Diagnosis and Treatment

Normal wound healing is a complicated process comprising hemostasis (begins immediately after injury), inflammation (starts shortly thereafter), proliferation (begins within days), and remodeling (lasts for several months to a year) (18, 21). The healing wound attains about 40% of its final strength in the first month, and the wound strength continues to increase for as long as a year after injury, but multiple factors affect and interfere with the healing process. Different types of sutures have different complete absorption time, meaning they support wound healing for different periods (22). The tensile strength of sutures, suturing approach, and surgeon’s experience may also affect the reliability of the closure, meaning that complete vaginal cuff healing varies. Early resumption of intercourse may be associated with incomplete vaginal cuff healing, especially in young adults (23).

VCD(E) typically occurs within the first 3 months postoperatively, but sporadic cases of VCD(E) have been historically reported as early as 3 days (24) and as late as 30 years (25) after TH. Consistent with published literature, the median TH-to-VCD(E) interval was 3.13 months in our study; half of the patients experienced VCD(E) within 3 months. The recommended time to resume sexual activities after TH was approximately 8 (13, 26) to 12 weeks (26) in the literature. Based on our experience, we suggest that patients resume normal sexual activities at least 3–6 months after TH.

A gynecologist should inform patients about the risk of VCD(E) after TH. Once patients present with symptoms related to VCD(E), medical advice should be provided at once. On the other hand, if patients present with complaints after TH, doctors should promptly conduct a medical history and physical examination. VCD(E) is a clinical diagnosis (13, 27) that should be made based on a history of TH and vaginal examination. Notably, people who underwent TH for obstetrical reasons could also experience VCD(E) (28). Early recognition of VCD(E) is imperative as it allows clinicians to implement effective treatments and is critical to preventing serious complications. In our study, most patients visited hospitals promptly as they experienced discomfort and were well managed immediately.

The therapeutic regimen should be tailored to each patient’s condition and surgeon’s judgment. On speculum and abdominal examination, if patients are diagnosed with VCD(E) and without evidence of secondary injury and peritonitis, efforts should be made to reduce the eviscerated organs as soon as possible. In our case series, after noting that the herniated bowel was ruddy and peristalsis was good, the protruding bowels of 4 of 11 VCDE patients were smoothly repositioned to the peritoneal cavity with a warm-wet gauze pad without anesthesia in the emergency department. Vaginal bowel reduction has been reported in several studies (5, 29, 30); however, our study may be the first to propose repositioning of prolapsed organs without anesthesia in the emergency department. Although manual bowel reduction without anesthesia may be slightly painful for patients, it still has numerous benefits. First, it can immediately free the patients from bulges. Second, it can provide a clear visualization for subsequent transvaginal procedures. Finally, it can reduce the likelihood of ischemia and necrosis of the eviscerated organs to some extent.

Conservative treatment can be an alternative option when a patient is medically stable with a partial VCD and without evisceration. It mainly involves hemostasis (hemostatic and local compression), activity restriction, and prophylactic antibiotics. Zoë Boersen et al. (1) reported two cases that were successfully managed conservatively. Notably, if the patient’s condition worsens, surgical intervention should be considered. Emergency cuff closure is mandatory in most cases. To date, no consensus has been reached regarding the optimal approach to separate cuff closure. Historically, VCD(E) was an indication for emergency surgery and suggested laparotomy (4). Subsequently, various techniques have been introduced (13), such as the transvaginal, laparoscopic, and combined abdominal-vaginal or laparoscopic-vaginal approaches. In our institution, 11 patients underwent a transvaginal approach, 2 patients underwent a combined laparoscopic-vaginal approach, and 1 patient underwent a laparoscopic approach. In case No.1, the serosal surface of the sigmoid colon adhered to the vaginal stump and continued with the top of the anterior vaginal wall. To decompress adhesion and repair the vaginal stump with good visualization, gynecologists converted from a transvaginal approach to a laparoscopic approach. A combined laparoscopic-vaginal approach was adopted in the other two, both of which underwent VCC. In case No.10, laparoscopy was performed at the end of VCC to confirm the integrity of the bladder using the methylene blue test. In case No.14, gynecologists conducted laparoscopy to resect a part of the necrotic omentum and bilateral ovaries preserved in the previous surgery. Importantly, the treatment strategy should be patient-specific. In medically stable patients who are free of any complications, vaginal repair is appropriate (5); it is economically prudent and avoids an extra surgical incision. In the case of women with a very narrow vagina or massive obesity (31), transvaginal procedures are challenging because of the limited visual fields. Laparoscopy has more advantages for recurrent VCD. Gerard-Peter reported a technique for laparoscopic repair using an omental flap (32). This approach allows the inspection of the peritoneal cavity and improves poor tissue vascularity. Certainly, when there is a need to excise the necrotic bowel or when there is a suspicion of peritonitis or intraperitoneal hematoma, an abdominal approach is appropriate (32, 33).

### Other Interventions

Prophylactic antibiotics should be administered to patients with VCD(E) to prevent infections (32). For hemodynamically unstable patients, timely and aggressive fluid resuscitation and blood transfusion play a vital role in prognosis. One patient in our cohort progressed to hemorrhagic shock and received a blood transfusion containing 10 units of red cell suspension and 1,200 mL fresh frozen plasma intraoperatively. Finally, the patient recovered well. Intraoperative placement of a vaginal drainage tube is essential when patients present with heavy vaginal discharge. If there is any possibility of bladder injury, creatinine level of the discharge or methylene blue test could be used to verify the integrity of the bladder. Notably, these two methods are simple and less invasive than cystoscopy. To restore well-vascularized tissue, debridement of the necrotic tissue of the vaginal cuff is necessary (34). Pathological examination was conducted in one patient in our cohort, revealing no particular lesions other than acute and chronic inflammation, similar to previous reports (10, 34). Jennifer K (18) compared the vaginal cuff specimens of VCD(E) with those of non-VCD(E) patients and found that VCD demonstrated dramatically higher levels of inflammatory cells. A prolonged inflammatory phase was reported to possibly delay the healing of the vaginal cuff because it slows normal progression to the reparative state and collagen remodeling (18). Considering these findings, pathological examination of the excised cuff tissue is valuable and will be essential for exploring the relationship between inflammation and VCD(E) in the future. Multidisciplinary cooperation also plays a key role in the management of VCD(E). It primarily involves the General Surgical Department, Blood Transfusion Department, Emergency Operating Room, and Anesthesiology Department.

The strength of the present study is that it shares our unique experience and suggests new, less invasive and individualized treatment approaches for VCD(E). Considering the extremely low incidence of VCD(E) and mature surgical techniques for TH, prompt management bears more importance than the prevention of VCD(E). The limitations of this study are consistent with those of previous studies. Our study was retrospective, and although the sample size was large, it is still limited. A large number of cases and a longer follow-up period in future studies are needed to validate the utility of our suggestions.

## Conclusion

LH is a risk factor for VCD(E), and early resumption of sex after TH is the most common trigger of VCD(E). Clinicians should educate patients to postpone coitus for at least 3–6 months after TH. Timely medical attention and patient-specific surgical management are crucial to avoid serious complications. Manual reduction of eviscerated organs without anesthesia in the emergency department, along with VCC, is feasible and minimally invasive.

## Data Availability

The original contributions presented in the study are included in the article/Supplementary Material, further inquiries can be directed to the corresponding author/s.
